# Proteome-wide Mendelian randomization and colocalization analysis identify therapeutic targets for cutaneous melanoma

**DOI:** 10.1097/MD.0000000000044678

**Published:** 2025-09-19

**Authors:** Di Zhou, Wenrong Luo, Jiefeng Zou, Xiaohai Zhu, Zheyuan Hu, Xiang Jie, Lie Zhu, Minjuan Wu

**Affiliations:** a Department of Burns and Plastic Surgery, the second affiliated hospital of Naval Medical University, Shanghai, China; b Department of Histology and Embryology, Naval Medical University, Shanghai, China.

**Keywords:** colocalization analysis, cutaneous melanoma, Mendelian randomization, plasma proteins, therapeutic targets

## Abstract

Cutaneous melanoma (CM) is the deadliest form of skin cancer, posing a significant threat to human health. In the ongoing efforts to develop effective treatments, identifying novel therapeutic targets is crucial. This research aimed to use Mendelian randomization and colocalization analysis to discover plasma proteins that could serve as new therapeutic targets for CM, while also assessing the potential adverse effects associated with these targets. The study harnessed plasma protein data from the UK Biobank Pharmaceutical Proteomics Project database, which contained genome-wide association data for 2940 proteins. These data were integrated with CM data from the Finnish database, involving 3194 patients and 314,193 controls. Proteome-wide analysis was then conducted to explore the associations between plasma proteins and CM risk. Through the proteome-wide analysis, 2 proteins, tyrosinase-related protein 1 and dipeptidase 1, were identified to have significant associations with CM risk. Notably, dipeptidase 1 exhibited an inverse relationship with CM risk, indicating its potential as a therapeutic target. However, the findings also raised concerns about its possible link to dementia. This comprehensive research approach successfully illuminated the causal relationships between specific plasma proteins and CM. It not only identified potential therapeutic targets but also emphasized the importance of understanding the broader implications of targeting these proteins, including potential adverse effects. The results lay the groundwork for further exploration of personalized treatment strategies for CM.

## 1. Introduction

**Figure 1.**
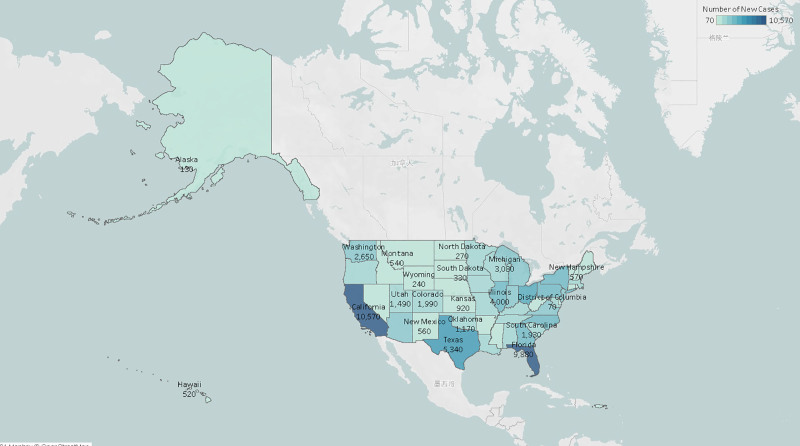
Number of new cutaneous melanoma patients in U.S. states in 2024.

Cutaneous melanoma (CM), the deadliest form of skin cancer, originates from malignant transformation of melanocytes.^[1]^ Its incidence is steadily rising worldwide, posing a pressing public health challenge. An estimated 100,640 new CM cases are projected in the United States for 2024, with state-specific incidence data presented in Figure 1 and Table S1 (Supplemental Digital Content, https://links.lww.com/MD/Q75).^[2]^ Despite significant advances in elucidating its genetic basis – including the identification of key oncogenes such as CDKN2A, MC1R, and N-Ras – prognosis for advanced-stage disease remains poor.^[3–5]^ While targeted therapies and immune checkpoint inhibitors have improved outcomes for subsets of patients, a critical unmet need persists for novel therapeutic options, particularly for resistant or refractory CM.

Against this backdrop, interest in utilizing proteome-wide association studies to characterize protein alterations and their genetic determinants in CM has grown. Unlike genes or RNA, proteins mediate most cellular functions and represent primary therapeutic targets. Existing evidence links CM initiation, progression, and metastasis to diverse circulating proteins; notably, premelanosome protein, CD38, monoacylglycerol lipase, and tumor necrosis factor-related apoptosis-inducing ligand.^[6–9]^ However, known associations between plasma proteins and CM, derived from observational studies or gene microarrays, are susceptible to confounding and reverse causality. Furthermore, ethical constraints preclude conducting randomized controlled trials to explore causal relationships between thousands of plasma proteins and CM. Proteome-wide Mendelian randomization (MR) offers a robust analytical framework by leveraging the natural randomization of genetic variants to infer protein–disease causal relationships, thereby circumventing the confounding and reverse causality inherent in traditional observational studies.

In this study, we employed proteome-wide MR analysis, leveraging extensive genetic and proteomic data from a CM cohort, to systematically screen for proteins with potential as therapeutic targets for CM. Subsequently, colocalization analysis was used to mitigate confounding bias arising from linkage disequilibrium (LD) between genetic variants associated with the target proteins and CM. Finally, Phenotype-wide association study (PheWAS) was performed to explore whether these proteins, when considered as therapeutic targets for CM, are associated with other phenotypes (potential adverse reactions). Through these analyses, we aimed to identify novel therapeutic targets for CM and provide new options for its clinical management.

## 2. Methods

### 2.1. Study design and ethics

**Figure 2.**
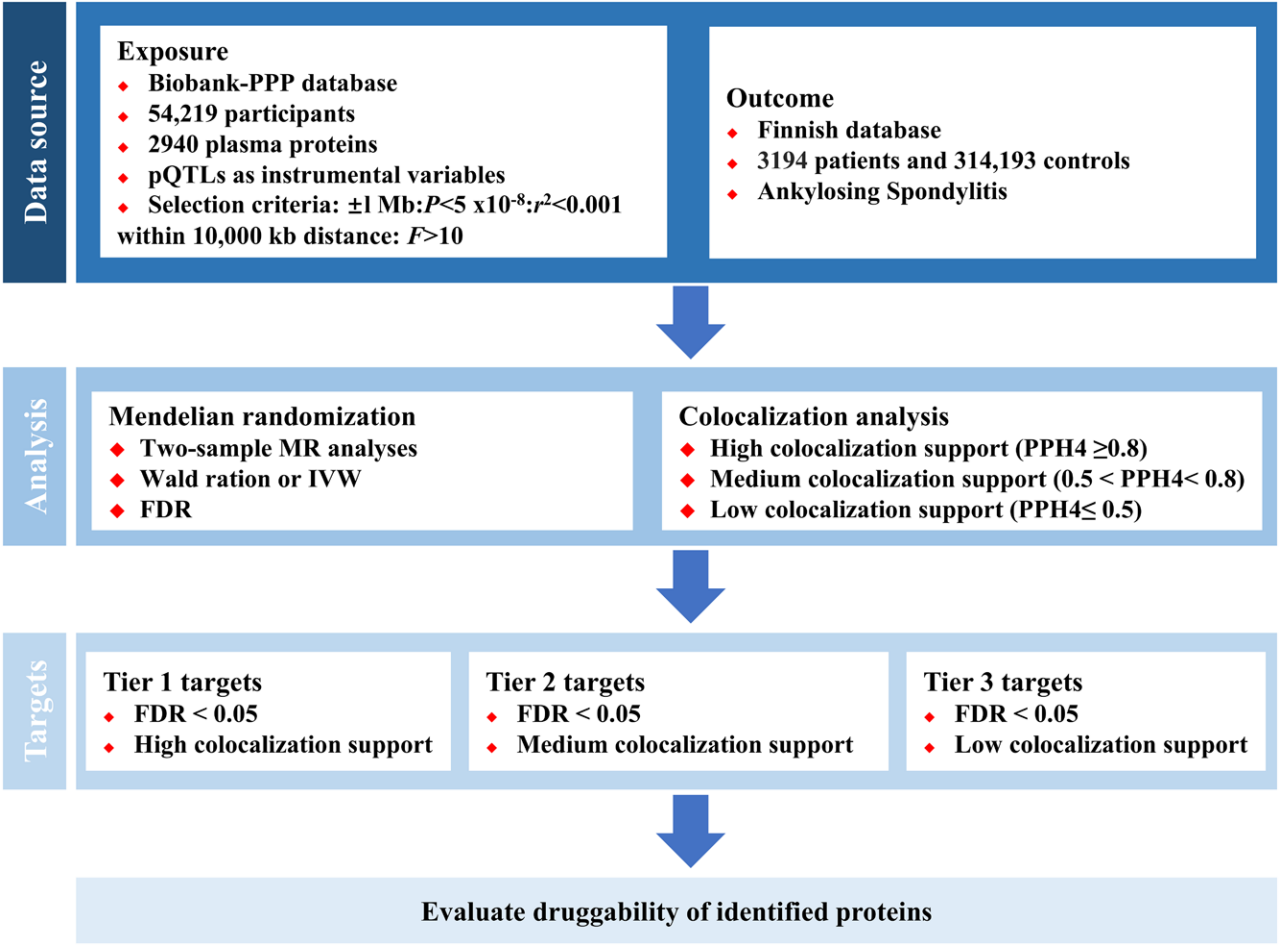
Flow chart of proteome-wide Mendelian randomization and colocalization analysis. Bio-PPP = Biobank Pharmaceutical Proteomics Project, FDR = false discovery rate, IVW = inverse variance weighting, pQTLs = protein quantitative trait loci.

Our study workflow is depicted in Figure 2. Data were derived from aggregated Genome-Wide Association Study (GWAS) datasets, which originated from original studies deposited in public databases. Informed consent was obtained in the original studies prior to their publication. Notably, our analysis relied solely on summary-level statistics, thus rendering additional ethical approval unnecessary.

### 2.2. Proteomic data source

Protein quantitative trait loci (pQTLs) were extracted from the UK Biobank Pharmaceutical Proteomics Project database as the focal exposures. This dataset includes plasma proteomic profiles of 54,219 European participants, encompassing 2940 proteins (https://www.synapse.org/#!Synapse:syn51365303).^[10]^ In this study, pQTLs served as instrumental variables (IVs). To ensure the robustness and reliability of our findings, stringent criteria were applied for IVs selection: single nucleotide polymorphisms (SNPs) within ± 1 Mb of the target gene locus were selected; a genome-wide significance threshold of *P* < 5 × 10^−8^ was used to identify SNPs significantly associated with plasma proteins; to ensure independence of SNPs and minimize LD effects, a threshold of *r*²<0.001 (genetic distance: 10,000 kb) was applied; an *F*-statistic > 10 was used to exclude weak IVs.^[11]^

### 2.3. CM GWAS statistics

Genetic association data for CM were obtained from the Finnish database (version R10; https://r10.finngen.fi/), which included 3194 cases and 314,193 controls.

### 2.4. MR analysis

MR is a robust methodology for inferring causal relationships between exposures and outcomes, using genetic variants as IVs. Here, we performed MR analyses to explore the causal association between plasma proteins (exposure) and CM (outcome). MR relies on 3 key assumptions for valid causal inference: IVs (pQTLs herein) are strongly associated with the exposure (plasma proteins); IVs are not associated with confounders influencing both plasma proteins and CM; IVs affect CM exclusively through their influence on plasma proteins (no horizontal pleiotropy).

For MR analyses, the Wald ratio method was used for proteins mapped to a single SNP, and the inverse variance weighting (IVW) method for those associated with multiple SNPs.^[12]^ We validated each assumption as follows: Assumption 1: Stringent pQTLs selection criteria were applied – only SNPs associated with plasma proteins at genome-wide significance (*P* < 5 × 10^−8^) and with *F*-statistics > 10 were included – to ensure strong IVs-exposure associations and minimize bias from weak instruments. Assumption 2: SNPs in LD (*r*²<0.001 within 10,000 kb) were excluded to ensure genetic independence, reducing the likelihood of IVs associating with unmeasured confounders. Assumption 3: Horizontal pleiotropy was evaluated using the MR-Egger regression intercept; a significant intercept (*P* < .05) indicated potential pleiotropy, which was addressed via sensitivity analyses. Heterogeneity was assessed using Cochrane *Q* statistic; significant heterogeneity (*P* < .05) was similarly evaluated with complementary methods to ensure robustness.^[13]^ Additionally, the false discovery rate (FDR) was used to control for multiple comparisons, with a threshold of FDR < 0.05.^[14]^

### 2.5. Colocalization analysis

Colocalization analysis is a pivotal method to identify shared causal genetic variants between exposure and outcome, thereby mitigating confounding from LD or other factors. For proteins with significant MR associations, we performed colocalization analysis using the *coloc* R package to determine whether their observed associations with CM were driven by LD effects. Five hypotheses were formulated prior to analysis: H0 indicates that the SNPs within the selected loci were not related to either the protein or CM; H1 indicates that SNPs associated with the protein but not CM, but not disease B; H2 indicates that SNPs associated with the protein but not CM; H3 indicates that SNPs associated with both the protein and CM, but via distinct causal variants. H4 indicates SNPs associated with both the protein and CM via the same causal variant.^[14]^ Posterior probabilities (PP) for each hypothesis were calculated. Colocalization evidence was categorized based on PPH4 (posterior probability for H4) as low (PPH4 ≤ .5), medium (.5 < PPH4 < .8), or high (PPH4 ≥ .8).

### 2.6. PheWAS

PheWAS, often referred to as “reverse GWAS”, investigates potential adverse effects of drug targets by examining associations between genetic variants (or phenotypes) and a broad range of other phenotypic traits. In the current study, plasma proteins with significant MR associations were treated as exposures, while outcomes included 2408 phenotypic traits from the Finnish database (version R10). A phenome-wide MR analysis was performed using this dataset, with statistical significance defined as FDR < 0.05.

### 2.7. Statistical analysis

Statistical analyses were performed using the R package “Two-Sample-MR” (version 0.5.6; MRC Integrative Epidemiology Unit [MRC IEU], Bristol, England, United Kingdom). For MR analyses, IVW was used for proteins with ≥ 2 SNPs (aggregating SNP effects via IVW) and the Wald ratio for single SNP. Horizontal pleiotropy was evaluated via MR-Egger regression (intercept *P* < .05 indicating significance), and heterogeneity via Cochran *Q* statistic. Multiple comparisons were corrected using FDR (threshold < 0.05). Colocalization analyses assessed shared causal variants via PP for 5 hypotheses, with PPH4 ≥ .8 indicating strong colocalization. PheWAS used FDR < 0.05 to define significant associations with 2408 phenotypes.

## 3. Results

### 3.1. Plasma proteins associated with CM

After screening with the most stringent thresholds, 1927 plasma proteins were included in the MR analysis (detailed in Table S2, Supplemental Digital Content, https://links.lww.com/MD/Q75). Using these proteins as exposures and their corresponding SNPs as IVs, a two-sample MR analysis was performed to assess associations with CM (outcome). For proteins with multiple SNPs, the IVW method was used; for those with a single SNP, the Wald ratio method was applied. Multiple comparisons were corrected using FDR.

**Figure 3.**
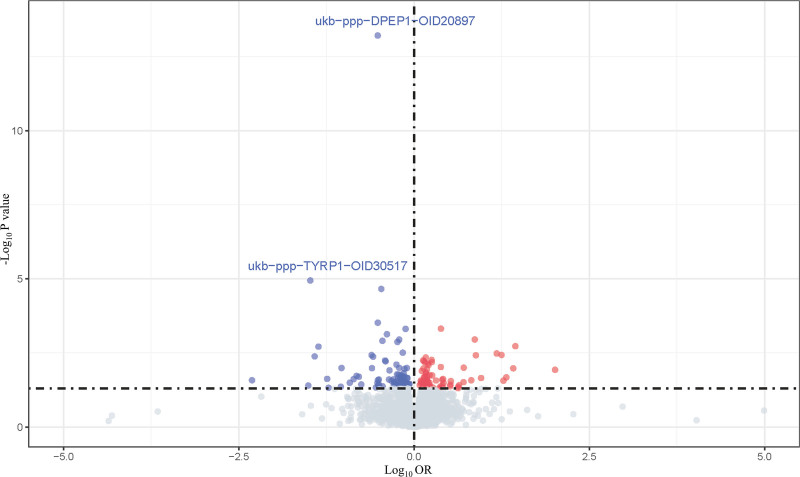
Volcano plot visualizing proteome-wide MR associations between plasma proteins and CM. The *x*-axis is labeled “log_2_ (OR),” representing the direction/magnitude of causal associations. The *y*-axis is “−log_10_ (*P* value),” reflecting statistical significance of *P* value. (Statistical analyses: Associations were estimated using IVW for proteins with multiple SNPs and Wald ratio for single SNPs; *P* < .05 was considered significant.) (Gray dots represent no significant differences, red dots indicate positive effect strength, and blue dots denote negative effect strength. Following FDR correction, TYRP1 and DPEP1 exhibit a *P* value > .05). CM = cutaneous melanoma, DPEP1 = dipeptidase 1, FDR = false discovery rate, IVW = inverse variance weighting, MR = Mendelian randomization, OR = odds ratio, SNPs = single nucleotide polymorphisms, TYRP1 = tyrosinase-related protein 1.

**Figure 4.**
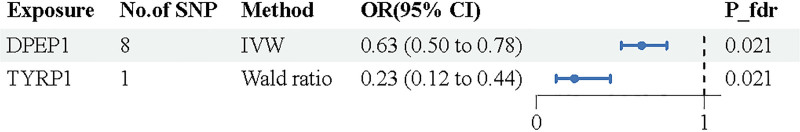
The results of MR analysis. MR = Mendelian randomization.

After FDR correction, 128 proteins showed potential associations with CM (Table S3, Supplemental Digital Content, https://links.lww.com/MD/Q75), but only tyrosinase-related protein 1 (TYRP1) and dipeptidase 1 (DPEP1) reached statistical significance with negative associations (no proteins had significant positive associations) (Fig. 3). Specifically, TYRP1 (OR: 0.23, 95% confidence interval (CI): 0.12–0.44, FDR: 0.02) and DPEP1 (OR: 0.63, 95% CI: 0.50–0.78, FDR: 0.02) were negatively associated with CM (Fig. 4).

TYRP1, instrumented by a single SNP, was analyzed via the Wald ratio method (*P* < .01). DPEP1, instrumented by multiple SNPs, was analyzed via IVW method (*P* < .01). For DPEP1, MR assumptions were validated: the MR-Egger intercept test showed no horizontal pleiotropy (*P* for Egger intercept > .05), supporting IVW as the primary result. Cochran *Q* test indicated significant heterogeneity (*Q* = 18.67, *P* < .01), but leave-one-out analysis revealed no drastic fluctuations in effect estimates after excluding individual SNPs. This suggests heterogeneity stems from accumulated effect differences among SNPs or natural variations in the exposure’s multi-pathway influence on CM, rather than bias from a specific SNP. Forest plots, scatter plots, and leave-one-out plots of the MR analysis results for DPEP1 and CM are shown in Figure S1 (Supplemental Digital Content, https://links.lww.com/MD/Q74).

### 3.2. Candidate therapeutic targets for CM identified by colocalization

To further validate their potential association with CM, we performed colocalization analysis on the 2 proteins identified as negatively associated in the MR analysis (Tables S4 and S5, Supplemental Digital Content, https://links.lww.com/MD/Q75).

The results demonstrated a significant colocalization effect of rs12447902 in DPEP1 and CM (PPH4 = 1), confirming the presence of substantial colocalization. As a consequence, we categorized DPEP1 as a Tier 1 target. For TYRP1, the most significant signal (rs10809826) had a low PPH4 (.14), below the medium evidence threshold (.5 < PPH4 < .8) and contrasting with DPEP1 (PPH4 = 1, meeting the high evidence threshold of PPH4 ≥ .8). This indicates weak colocalization, suggesting the observed association with CM is unlikely driven by shared causal genetic variants (genetic signals may overlap coincidentally rather than causally). TYRP1 was therefore classified as a Tier 3 target, with reduced confidence as a prioritized therapeutic target.

**Figure 5.**
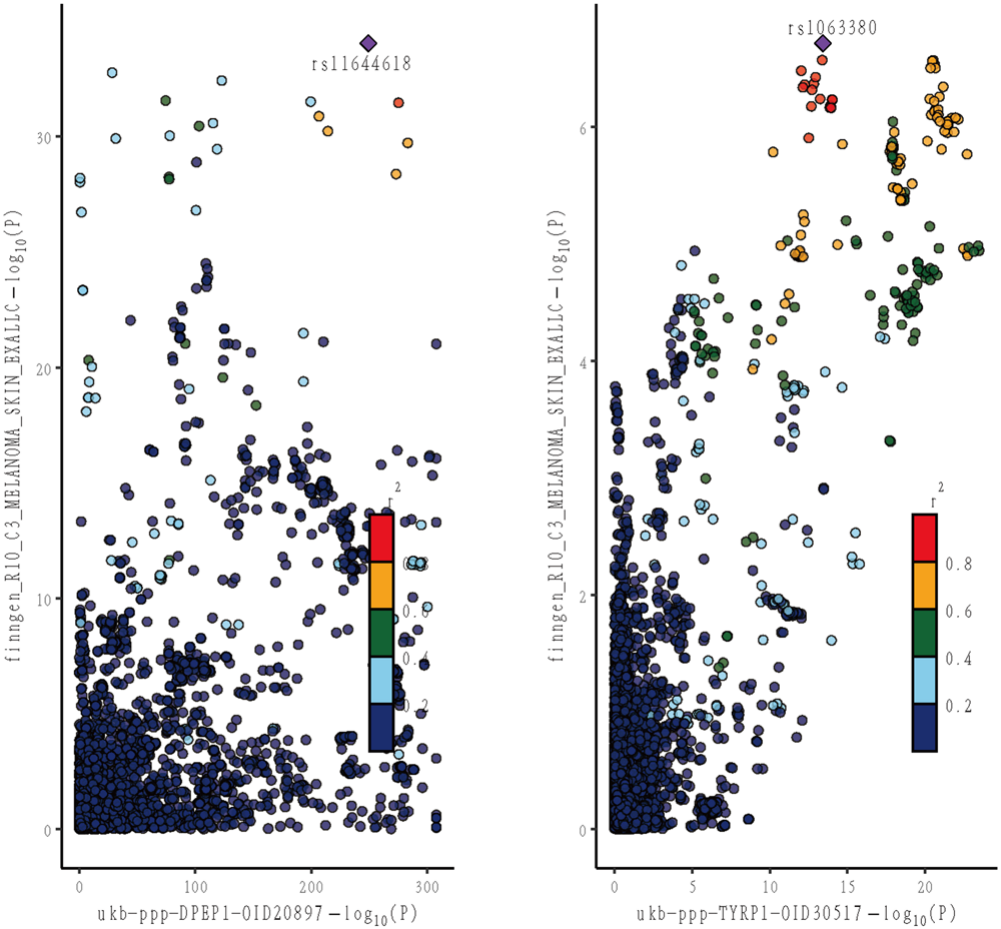
Results of colocalization analysis of proteins with positive results of MR analysis. MR = Mendelian randomization.

Colocalization results are shown in Figure 5. Considering the strength of colocalization evidence, DPEP1 may be a more efficacious therapeutic target for CM than TYRP1.

### 3.3. PheWAS for 2 plasma proteins linked to CM

**Figure 6.**
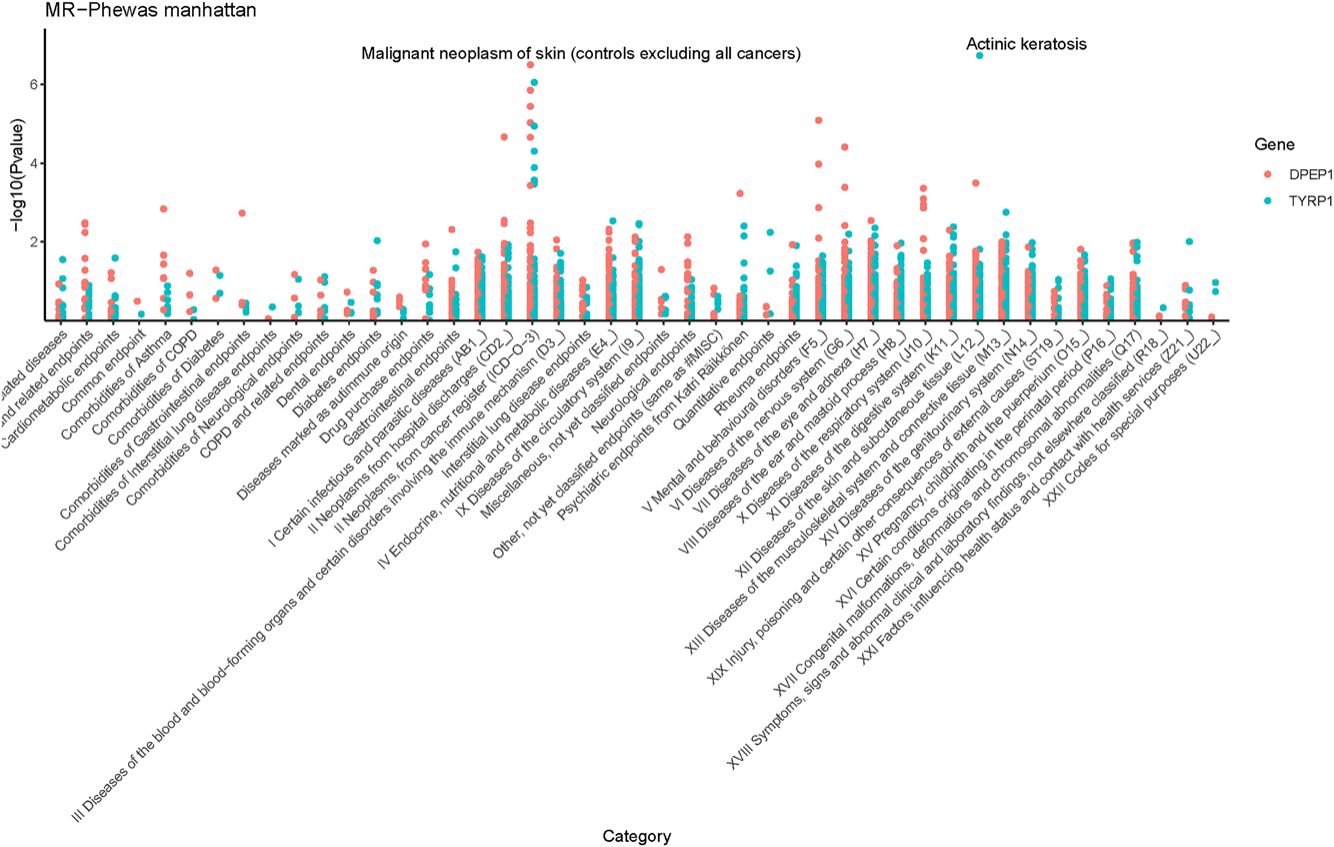
Results of phenome-wide association study of proteins with positive results of MR analysis. MR = Mendelian randomization.

To explore potential roles of DPEP1 and TYRP1 in other phenotypes, we conducted PheWAS using these proteins as exposures and 2408 phenotypes from the Finnish database (version R10) as outcomes (Fig. 6). DPEP1 was associated with dementia and other nonmelanoma skin cancers, while TYRP1 was associated with actinic keratosis (AK) (Tables S6 and S7, Supplemental Digital Content, https://links.lww.com/MD/Q75).

## 4. Discussion

CM is characterized by aberrant transformation and uncontrolled proliferation of melanocytes in the epidermal basal layer, resulting in malignant skin tumor formation.^[15]^ Susceptibility to CM is influenced by multiple risk factors, including sun exposure, indoor tanning, family history, and numerous nevi. While CM can affect individuals across all age groups, its incidence rises significantly with age, particularly in older adults.^[16]^ CM is more aggressive than other skin malignancies (e.g., squamous cell carcinoma and basal cell carcinoma), primarily due to its high metastatic potential.^[17]^ It typically metastasizes initially via lymphatic spread in early stages, progressing to hematogenous dissemination as the disease advances; the lungs are the most common target of metastatic lesions. Notably, early-stage CM (stages I–II) has a favorable prognosis and is often curable with complete surgical resection, with an excellent 5-year survival rate of ~99.4%. However, prognosis deteriorates drastically in advanced stages (stages III–IV): stage III CM has a 5-year survival rate of only 68%, and stage IV drops to < 30%.^[18]^ The development of targeted therapies and immune checkpoint inhibitors has transformed the management of unresectable CM, substantially improving patient survival. Traditional targeted therapies primarily target the BRAF and MEK pathways, with drugs such as vemurafenib, dabrafenib, and trametinib playing key roles in clinical practice.^[19–21]^ Nevertheless, despite initial favorable responses, prolonged treatment often leads to drug resistance – a major clinical challenge. Thus, there is an urgent need to identify novel, stable, safe, and efficacious therapeutic targets for CM management.^[1]^

MR analysis uses genetic variants associated with an exposure to evaluate potential causal relationships between the exposure and outcomes, aiming to mitigate biases from confounding and reverse causation inherent in observational studies. In this study, two-sample MR analysis was employed to identify potential therapeutic targets for CM. We found that DPEP1 and TYRP1 were negatively associated with CM, supporting their potential as therapeutic targets. Validation of core MR assumptions confirmed that our findings for DPEP1 and TYRP1 are robust: no evidence of horizontal pleiotropy or significant heterogeneity was detected. These results reinforce that the observed associations are likely causal rather than confounded.

The gene encoding DPEP1 is localized to the q24 band of human chromosome 16.^[22]^ As a zinc-dependent metalloproteinase, DPEP1 degrades excess peptides and antibiotics (e.g., thienamycin, penems, and carbapenem derivatives) and plays a critical role in glutathione and leukotriene metabolism.^[23]^ DPEP1 has been linked to various tumors: it serves as a reliable marker for high-grade intraepithelial neoplasia and colorectal cancer, with potential applications in early tumor detection and prognostic stratification.^[24,25]^ For hepatocellular carcinoma (HCC), DPEP1 enhances diagnostic efficacy when combined with alpha-fetoprotein (AFP). Alone, DPEP1 has a diagnostic accuracy of 0.75 (95% CI: 0.68–0.83) for HCC, with sensitivity and specificity of 78.79% and 62.5%, respectively; in combination with AFP, accuracy increases to 0.82 (95% CI: 0.75–0.89). Notably, in AFP-negative HCC patients, DPEP1 maintains a diagnostic accuracy of 0.79 (95% CI: 0.71–0.88; sensitivity 73.68%, specificity 68.75%) and effectively discriminates small HCC lesions (<3 cm in diameter) with an accuracy of 0.76 (95% CI: 0.64–0.88).^[26]^ Beyond oncology, DPEP1 functions as the primary adhesion receptor for neutrophils, orchestrating leukocyte recruitment during pulmonary inflammation and thereby exacerbating tissue damage in endotoxemia.^[27]^

TYRP1 is specifically expressed in melanocytes and melanoma cells.^[28]^ Also referred to as 5,6-dihydroxyindole-2-carboxylate oxidase or gp75 glycoprotein in some studies, it acts as a key enzyme in melanin biosynthesis: it catalyzes the oxidation of tyrosine to 5,6-dihydroxyindole-2-carboxylic acid – a critical intermediate in the pathway – thereby promoting melanin synthesis.^[29,30]^ As a natural pigment, melanin is essential for skin, hair, and eye pigmentation; TYRP1 enzymatic activity regulates melanin production, thus influencing tissue pigmentation. Genetic and environmental factors can modulate TYRP1 activity, highlighting its pivotal role in regulating skin and hair pigmentation. In an open-label, dose-escalation phase I study, 27 patients with advanced melanoma (who had progressed on or during at least 1 prior therapy) received intravenous IMC-20D7S (a recombinant human IgG1 monoclonal antibody targeting TYRP1) every 2 or 3 weeks. One patient achieved a complete response, with a disease control rate (stable disease or better) of 41%.^[31]^ These findings support TYRP1 as a promising therapeutic candidate for CM, though larger sample sizes and longer follow-up are needed to fully evaluate its safety and efficacy. Notably, this aligns with our MR analysis results, reinforcing the reliability of our findings. However, subsequent colocalization analysis revealed limited evidence for association between TYRP1 and CM, indicating that while TYRP1 is a therapeutic target for CM, its relationship with the disease is not driven by shared causal genetic variants.

Finally, we performed a PheWAS of DPEP1 and TYRP1 to explore potential side effects when these plasma proteins are considered as prospective therapeutic targets for CM (Tables S6 and S7, Supplemental Digital Content, https://links.lww.com/MD/Q75), with FDR adjustment applied to enhance result precision. Beyond its potential as a CM therapeutic target, DPEP1 was identified as a candidate for treating other nonmelanoma tumors (OR = 0.767, 95% CI: 0.693–0.849, FDR < 0.01), such as basal cell carcinoma. However, we note a potential association between DPEP1 and dementia (OR = 1.161, 95% CI: 1.087–1.240, FDR < 0.05), including Alzheimer disease. Proteomic analyses of cerebrospinal fluid from Parkinson disease patients with cognitive decline have shown altered DPEP1 expression compared to healthy controls.^[32]^ This association may be tenuous, as our DPEP1 data derive from plasma, while dementia involves central nervous system processes, and the underlying mechanism remains unclear. Thus, validation is critical: future studies should measure cerebrospinal fluid DPEP1, replicate in diverse cohorts, and explore pathways (e.g., neuroinflammation) to confirm clinical relevance. Nonetheless, vigilance regarding potential side effects in elderly populations – particularly those with dementia – is warranted during drug development. TYRP1 PheWAS results revealed a strong association with AK. AK is a chronic cutaneous condition characterized by clinical and subclinical lesions, predominantly on sun-exposed areas (head, neck, limbs),^[33]^ and is considered a precancerous lesion for keratinocyte carcinoma.^[34]^ Their association may involve ultraviolet-induced melanin alterations in the skin.

Known small-molecule inhibitors of DPEP1 (cilastatin and the LSALT peptide) may exert anti-CM effects, though no relevant studies have been reported to date.^[27,35]^ Additionally, analogous to enfortumab vedotin targeting Nectin4, anti-DPEP1 monoclonal antibodies for tumor-specific payload delivery may achieve targeted cytotoxicity against DPEP1-high melanoma cells, minimizing off-target effects.^[36]^ Combining DPEP1 inhibitors with BRAF/MEK inhibitors may also reduce drug resistance.^[37,38]^ These strategies hold promise for novel precision therapies in CM. As a melanocyte-specific antigen, TYRP1 offers multiple avenues for precision therapy development: 1. Anti-TYRP1 monoclonal antibodies showed a 41% disease control rate in phase I trials with favorable safety profiles, supporting potential in monotherapy or combinations;^[31]^ 2. TYRP1-directed chimeric antigen receptor T cells, optimized with a long flexible hinge and CD28 co-stimulation, exhibit potent efficacy against cutaneous and rare melanoma subtypes in preclinical models by targeting high surface TYRP1 expression;^[39]^ 3. Analogous to photodegradation-targeting chimeras, TYRP1-targeted photosensitizer conjugates encapsulated in nanoplatforms (e.g., via TYRP1 ligand-photosensitizer linkers) may enable tumor-specific spatiotemporal killing.^[40]^ These strategies leverage TYRP1’s restricted expression to enhance specificity and efficacy.

The strengths of our study include its comprehensive design: we analyzed a large panel of plasma proteins as exposures, utilized a substantial number of CM cases as outcomes, validated findings across 2 independent datasets, and supplemented results with colocalization analyses. Employing MR analysis mitigates confounding and reverse causality biases, thereby strengthening causal inference. Additionally, we performed a PheWAS to clarify the potential roles of target proteins. Nevertheless, several limitations should be noted. First, our analysis was predominantly based on European populations, restricting the generalizability of conclusions. Second, while we focused on the role of plasma proteins in CM, environmental factors – particularly ultraviolet radiation – are indisputably influential. Thus, integrating environmental exposures with plasma proteins in risk prediction models is warranted to better elucidate CM etiology. Finally, the lack of in vitro and in vivo experiments under physiological conditions, as well as randomized controlled trials, limits direct validation of our findings.

## 5. Conclusion

This proteome-wide MR and colocalization analysis identified 2 plasma proteins – DPEP1 and TYRP1 – that are causally associated with CM, with DPEP1 showing particularly strong evidence. These findings provide guidance and novel directions for targeted therapy development. We anticipate that these results will inform CM treatment strategies and may help alleviate the individual and societal burdens imposed by this disease.

## Author contributions

**Conceptualization:** Di Zhou, Xiang Jie.

**Funding acquisition:** Minjuan Wu.

**Investigation:** Di Zhou, Wenrong Luo.

**Methodology:** Zheyuan Hu, Lie Zhu.

**Resources:** Di Zhou, Jiefeng Zou.

**Supervision:** Xiaohai Zhu.

**Validation:** Di Zhou, Wenrong Luo, Xiang Jie.

**Writing – original draft:** Di Zhou, Wenrong Luo, Jiefeng Zou.

**Writing – review & editing:** Wenrong Luo, Minjuan Wu.

## Supplementary Material


