# Supplementary material for: Proteome-wide Mendelian randomization and colocalization analysis identify therapeutic targets for cutaneous melanoma
Source: Medicine (Baltimore). 2025 Sep 19;104(38):e44678. doi: 10.1097/MD.0000000000044678 (PMC12459469; doi:10.1097/MD.0000000000044678)
Supplement: Supplementary file 2 [file medi-104-e44678-s002.docx]

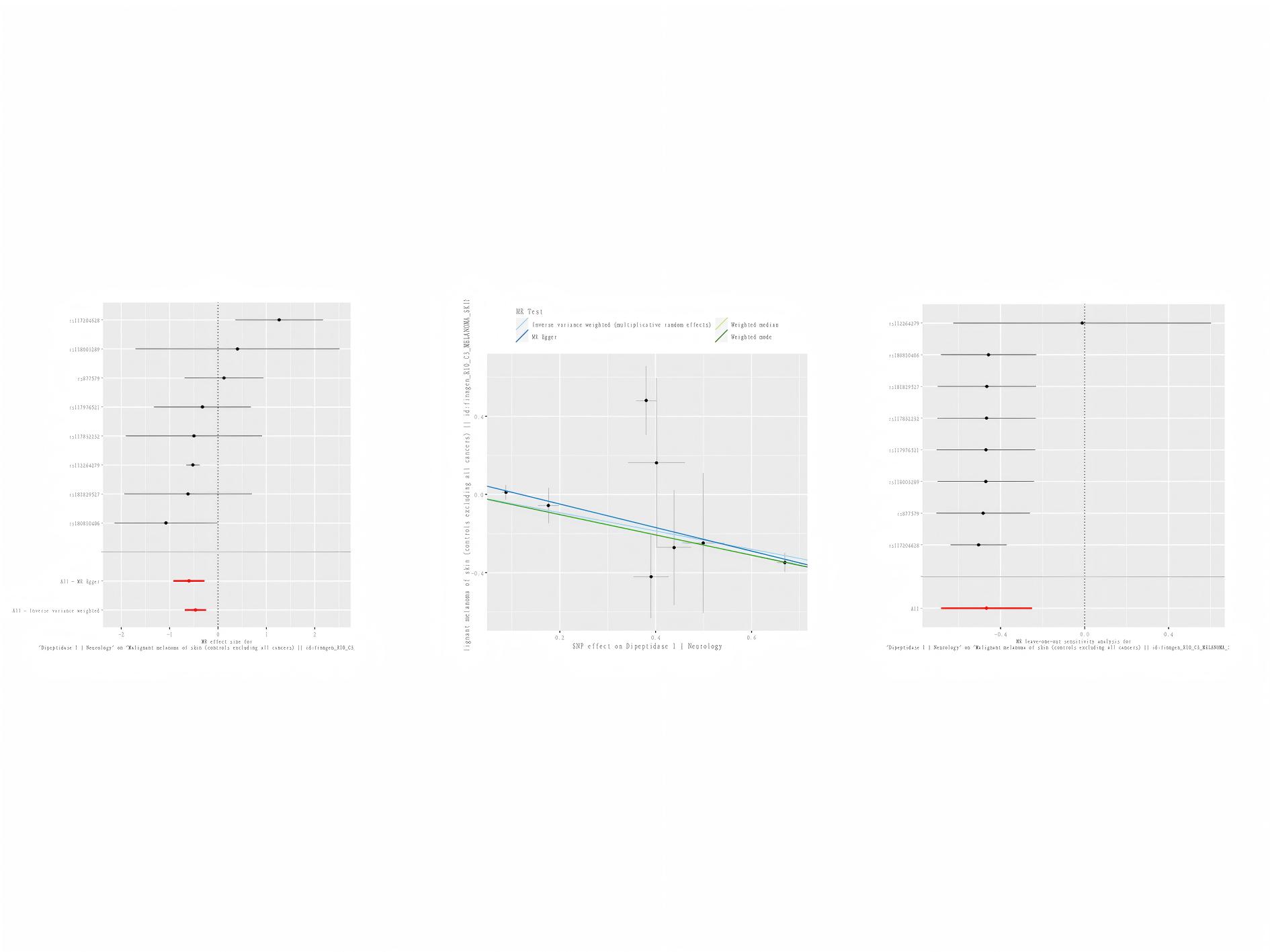


Supplementary Figure S1 Forest plots, scatter plots, and leave-one-out plots of the MR analysis results for DPEP1 and CM
